# Feasibility and Accuracy of a Computer-Assisted Self-Interviewing Instrument to Ascertain Prior Immunization With Human Papillomavirus Vaccine by Self-Report: Cross-Sectional Analysis

**DOI:** 10.2196/16487

**Published:** 2020-01-22

**Authors:** Carlos R Oliveira, Lital Avni-Singer, Geovanna Badaro, Erin L Sullivan, Sangini S Sheth, Eugene D Shapiro, Linda M Niccolai

**Affiliations:** 1 Section of Infectious Diseases and Global Health Department of Pediatrics Yale University School of Medicine New Haven, CT United States; 2 Department of Epidemiology of Microbial Diseases Yale University School of Public Health New Haven, CT United States; 3 Department of Obstetrics, Gynecology & Reproductive Sciences Yale University School of Medicine New Haven, CT United States

**Keywords:** human papillomavirus vaccine, self-report, accuracy, computer-assisted self-interviewing

## Abstract

**Background:**

Ascertaining history of prior immunization with human papillomavirus (HPV) vaccine can be challenging and resource-intensive. Computer-assisted self-interviewing instruments have the potential to address some of the challenges of self-reporting, and may also reduce the time, costs, and efforts associated with ascertaining immunization status.

**Objective:**

This study assesses both the feasibility and the accuracy of a computer-assisted self-interviewing instrument to ascertain a patient’s history of immunization with the HPV vaccine.

**Methods:**

We developed both a survey and a Web-based data collection system using computer-assisted self-interviewing to ascertain self-reported HPV vaccine immunization history. We implemented the instrument in a sample of adult women enrolled in an ongoing study of the HPV vaccine. Vaccine records from prior sources of care were reviewed to verify reported immunization history.

**Results:**

Among the 312 participants who provided HPV vaccine immunization history by self-report, almost all (99%) were able to do so using the computer-assisted self-interviewing instrument. The median survey completion time was 10 minutes (IQR 7-17). The accuracy of self-report was 84%, sensitivity was 89%, specificity was 80%, and the negative predictive value was 92%.

**Conclusions:**

We found that it is feasible to collect a history of immunization with the HPV vaccine using a computer-assisted self-interviewing instrument. This approach is likely to be acceptable to adult women and is reasonably accurate in a clinical research setting.

## Introduction

Highly efficacious vaccines against human papillomavirus (HPV) have been available in the United States to prevent cervical cancer and its precursors since 2006 [1]. These vaccines are recommended for females between the ages of 11-26 years old and for males between the ages of 11-21 years old. Although immunization in early adolescence is ideal, many young adults (18-26 years old) are unvaccinated and remain susceptible to developing cancer [2]. The lack of a readily available source of data for ascertaining prior immunization has been a significant barrier to the study of the HPV vaccine in this population [3,4]. Vaccine records are often incomplete or scattered among numerous sites, making efforts to ascertain prior immunization by reviewing vaccine records a lengthy and labor-intensive process [5]. Hence, researchers and clinicians often find it more practical to rely on a patient’s self-reporting to ascertain HPV vaccine immunization status [6-9]. However, little has been done to establish the validity of self-reporting in this context.

Computerized data collection systems have been increasingly used in clinical research to reduce both the burden and the inaccuracies associated with manual data entry. Computer-assisted self-interviewing methodologies are an extension of these data-collection systems. They have been found to be useful for eliciting more candid responses when the information requested is perceived as either private or too sensitive to disclose in-person [10,11]. Additionally, studies have shown that computer-assisted self-interviewing may remove the time-pressures to respond, which may improve the accuracy of reporting [12,13].

However, no previous studies have adapted computer-assisted self-interviewing methodologies for the assessment of immunization history. In this study, we describe the development of a new data collection instrument that uses computer-assisted self-interviewing methodologies to ascertain HPV vaccine immunization status by self-reporting among adult women. Additionally, we provide early results from our experiences implementing this instrument in a clinical research study.

## Methods

### Design of the Computer-Assisted Self-Interviewing Instrument

**Figure 1:**
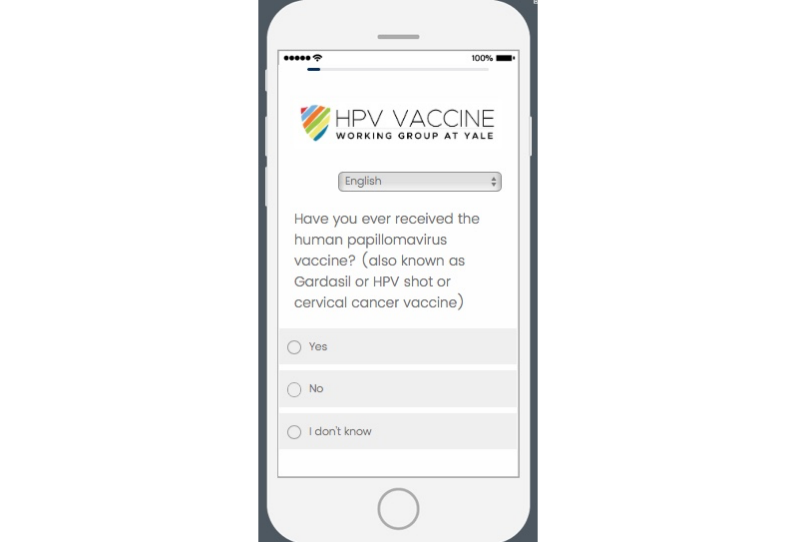
Representative screenshot of the app.

Using computer-assisted self-interviewing methodologies, we designed a Web-based data collection instrument aiming to reduce the time and resources needed to ascertain prior immunization with the HPV vaccine. The computer-assisted self-interviewing instrument was programmed using the Qualtrics Research Suite (Qualtrics LLC, Provo, Utah, United States) and was hosted on a secure Yale-Qualtrics server (approved for use with electronically protected health-information data) to allow participants to access the survey from any Web browser (including mobile devices) and to eliminate the need to download additional software. The graphical user interface (what the participant sees and uses) was designed to be both easy to use and intuitive, with clickable radio buttons and a simple presentation of questions (one at a time) to allow participants to control their pace fully. Survey questions were translated for Spanish speakers, and a dropdown menu was added to every page to allow respondents to change the language they wished to use for the survey at any time. The integrity of the data entered by the participants was ensured by incorporating several real-time data validation procedures, such as consistency checks and follow-up questions. A representative screenshot of the user interface is shown in Figure 1.

The questions in the survey were structured using an adaptive and modular format. The core module (a fixed set of questions displayed to all participants), requested information on prior immunization with the HPV vaccine, prior sources of medical care, and personal sociodemographic data. The secondary modules were adaptive and included follow-up questions that varied based on antecedent responses. For example, in the core module, all participants were asked if they had previously been immunized with the HPV vaccine; if the response to this question was “yes,” secondary modules that were specific to each dose received were added to the survey that inquired about the dates of immunization and the names/locations of their vaccine providers. Source code is available upon request, and survey questions can be found in Multimedia Appendix 1 (see Table A.1-2).

### Testing, Refinement, and Implementation of the Computer-Assisted Self-Interviewing Instrument

Before the deployment of the computer-assisted self-interviewing instrument, the prototype was tested in a sample of women who were representative of the future users (n=5) using the “think-aloud” method [14]. Participants were audio-recorded and asked to describe their experiences while completing the survey. Participants were also asked to comment on the flow, thematic design, readability, translation (if Spanish-speaking), and clarity of both the survey questions and instructions. Imprecise questions were modified, and suggested changes were incorporated into the user-interface after each interview until no further modifications were required.

As a final step, we implemented the computer-assisted self-interviewing instrument in a sample of adult women and conducted formal assessments of its feasibility and accuracy in a clinical research study. The sample for the computer-assisted self-interviewing implementation study was comprised of women aged 23-38 years old who had been recruited to participate in the HPV Vaccine Effectiveness (HPV-VE) Project [4], an ongoing, population-based, case-control study to determine the effectiveness of HPV vaccines against precancerous cervical dysplasia. A description of the case-control study, the inclusion criteria, and the study definitions are summarized in Textbox 1.

**Description of the HPV-VE Project.**

**HPV-VE Aims**

- A collaborative project between Yale University, the Connecticut Department of Public Health, and the Centers for Disease Control and Prevention, which aims to quantify the real-world effectiveness of HPV vaccines against high-grade cervical dysplasia attributable to HPV types 16 or 18.

**Eligibility**

- Women born during or after 1981.
- A resident of New Haven County, Connecticut, United States.
- Underwent screening for cervical cancer after January 1, 2010, in one of the clinics affiliated with the Yale New Haven Health System.

**Case**

- Diagnosed with a high-grade cervical lesion (cervical intraepithelial neoplasia grades two or higher).
- Positive test result from cervical lesion for HPV 16 or HPV 18.

**Matched Controls**

- Patients with normal cervical cytology.
- Matched to a case by age, gynecologic practice, and date of procedure to obtain a sample for cervical cytology.

All English- and Spanish-speaking women who were eligible and willing to participate in the HPV-VE Project were contacted by telephone and asked to complete a brief survey about their prior experiences with HPV vaccines and personal health. Women who were willing to complete the survey were given the option to do so either online, in-person, or using a mail-in survey. Subjects who opted to complete the survey online were granted access to the secure computer-assisted self-interviewing instrument via individualized, single-use links, and could enter their responses at the time and on the device of their choosing. Women who wished to participate in-person, and women who did not have access to either the internet or a personal computer/smartphone, were scheduled to complete the survey with research staff. During these scheduled appointments, investigators provided subjects with a touchscreen tablet, with the computer-assisted self-interviewing instrument preloaded, and gave them privacy to complete the survey independently. Research assistants were made available to clarify questions or to enter responses for subjects who preferred not to use the provided tablet. Study team members obtained written informed consent from all subjects before the distribution of our computer-assisted self-interviewing instrument. Screening and consent procedures were conducted by trained research staff using standardized scripts and in Spanish with women who were Spanish-only speakers. As a form of gratitude, a US $25 gift card was provided to participants after completion of the survey.

### Validation of Self-Report

Participants were asked to list all prior sources of medical care since 2006 when the vaccine was first made available in the United States. Contact information for listed prior sources of care was reviewed and updated as needed using Web searches and Yale-New Haven Health System directories. Medical practices were contacted by telephone, and appointments were scheduled for trained research staff to extract the participant’s immunization history on-site. If vaccine records were not available for on-site review, a copy of the signed consent form was sent to the medical practice with an extraction form to complete and return. Documentation of immunization by a medical provider was considered the gold standard for receipt of the vaccine. Immunization status was analyzed as a dichotomous variable based on whether the patient had ever received at least one dose of either the bivalent, quadrivalent, or nonavalent vaccine before completing the survey. A patient was considered “immunized by medical record” if documentation was found of at least one date of immunization on any vaccine record. A subject was considered “not-immunized by the medical record” if no date of immunization was found after reviewing all available records from the reported prior sources of care. A subject was considered “immunized by self-report” if they answered “yes” to the survey question “Have you ever received the human papillomavirus vaccine?” If the response was either “no” or “I don’t know,” they were considered “not immunized by self-report.”

### Analyses

Demographics and baseline patient characteristics are reported for both the eligible and enrolled groups. Logistic regression models were used to determine whether the eligible subjects who were willing to participate and provided a self-report differed from those who were invited but were unwilling to participate or did not provide self-report. The most recent zip code listed in the subject’s medical records was used as a proxy for socioeconomic status. This was accomplished by linking the subject’s zip code to the 2010 Census data [15], and determining if the subject lived in an area where there was either a low, medium, or high proportion of residents with incomes below the federal poverty threshold (10%, 11-19%, and ≥20% proportion below the poverty threshold, respectively), as has been previously described [16,17],

Diagnostic indices, including sensitivity, specificity, and positive and negative predictive values, were used to estimate the performance of self-report using computer-assisted self-interviewing compared with the immunization status in the records of all prior sources of care. Data generated by the Web browser being used to access the survey was collected to determine user preferences for data entry (mobile vs desktop device) and to capture timestamps for measures of efficiency. We assessed how participants used the survey by tabulating time from signed consent to starting the survey, time from starting the survey to completing it, and the proportion of participants who started the survey but did not complete all sections.

Secondary analyses determined whether the accuracy of self-report was associated with the participant’s sociodemographic characteristics or knowledge of the HPV vaccine. Knowledge of the HPV vaccine was estimated based on the number of correct responses to a series of true/false questions about HPV vaccine (see Multimedia Appendix 1, Table A.2). Among the participants who accurately recalled having been immunized (ie, participants for whom we were able to verify with medical records receipt of prior immunization), we estimated the accuracy of the reported number of doses received and the accuracy of the reported year of first immunization.

### Sensitivity Analyses

Sensitivity analyses were performed to assess the stability of the estimated accuracy of self-reported immunization status, including whether accuracy varied when the models were restricted to only cases, which were only matched controls or only subjects for whom all vaccine records could be reviewed. Statistical analyses were conducted using Stata statistical software 14.0 (StataCorp, College Station, Texas, United States). The institutional review board of Yale University approved this protocol.

## Results

### Overview

A total of 706 eligible subjects were invited to participate between January 2013 and December 2018, of whom 325 (46%) signed a consent form, and 312 (44%) provided self-report using the survey. The subjects who provided self-report were like those who were invited and did not provide self-report with respect to spoken language and area-based socioeconomic status (Table 1). *P* values were estimated using logistic regression and excluding missing/unknown observations. Area-based socioeconomic status was estimated using the subject’s zip code. Those categorized as unwilling to participate were women who declined, are undecided, or have yet to complete the survey.

**Table 1:** Willingness to participate and provide self-report of immunization history.

Demographics		Invited to participate (N=706)		
		Unwilling to participate	Provided self-report	*P* value
Total, n (%)		394 (56)	312 (44)	—^a^
Age (years), median (IQR)		33 (30-35)	32 (30-35)	.01
**Language, n (%)**				
	English	326 (83)	247 (79)	Reference
	Spanish	13 (3)	12 (4)	.63
	Other	15 (4)	13 (4)	.73
	Unknown	40 (10)	40 (13)	—
**Area-based socioeconomic status, n (%)**				
	Low poverty zip code	160 (41)	122 (39)	Reference
	Medium poverty zip code	97 (25)	60 (19)	.30
	High poverty zip code	137 (35)	130 (42)	.20

^a^Not applicable.

**Figure 2:**
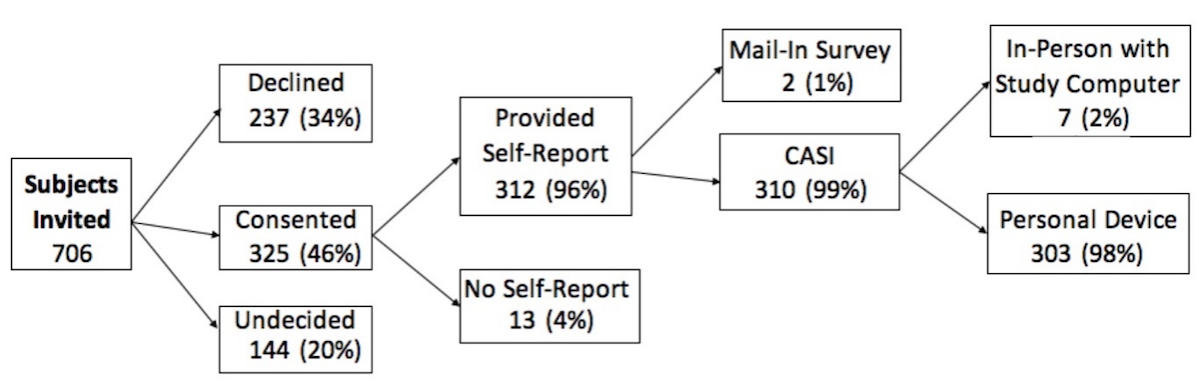
Enrollment flowsheet for CASI data collection instrument analyses. CASI: computer-assisted self-interviewing.

Among the 312 participants who provided a self-report, almost all (99%) were able to use the computer-assisted self-interviewing instrument, of which 303 (98%) opted to enter their responses using their device (Figure 2). Approximately 55% (n=169/312) of computer-assisted, self-interviewing users elected to access the survey on a mobile device. A few (n=7) asked to complete the survey in-person or needed the provision of a computer to access the survey. Only one participant asked for assistance in reading and entering responses into the tablet during the in-person interview. Two participants who opted to use computer-assisted self-interviewing on their device finished in an unusually short amount of time (bottom first percentile of the median survey completion time, which corresponds to <3 minutes). To avoid bias from survey satisficing, the self-report of these two individuals were not included in the computer-assisted self-interviewing performance analysis.

The median age of the computer-assisted self-interviewing users was 32 years old (IQR 30-35). Most had some college education (81%), spoke English (94%), and identified as White (57%) (Table 2). Public insurance consisted of Medicare, Medicaid, HUSKY, Indian Health Service, or military insurance. Due to completing their surveys too quickly, 2 participants were left out of the total user count.

**Table 2:** Characteristics of the study sample.

Demographics		CASI^a^ user (N=308)^b^
Age, median (IQR)		32 (30-35)
**Race/ethnicity, n (%)**		
	Non-Hispanic white	175 (57)
	Non-Hispanic black	54 (18)
	Hispanic	56 (18)
	Non-Hispanic other/multi-race	23 (7)
Publicly insured, n (%)		79 (26)
Some college education, n (%)		248 (81)
Annual income of <US $50,000, n (%)		102 (33)

^a^CASI: computer-assisted self-interviewing.

^b^Two participants excluded from total count due to completing the survey too quickly.

Self-reported immunization history was determined using computer-assisted self-interviewing at a median of 1 day (IQR 0-3) after consent. By comparison, the days elapsed between the investigator’s initial contact with the clinical staff of gynecology practices to the receipt of vaccine records was ax median of 5 days (IQR 0-14). The median time required for participants to finish the survey was 10 minutes (IQR 7-17). After reporting their immunization history, 5% (n=18/312) of participants opted not to answer some or all the remaining survey questions.

A total of 780 vaccine records were reviewed for the 312 participants who provided a self-report. Vaccine records from at least one reported source of care were reviewed for every subject (mean of 2 sources of care were reviewed per subject). Receipt of at least one dose was documented in the medical records for 39% (n=122/780) of participants. Receipt of three or more doses was documented for 27% (n=85/780). Of the 307 vaccine doses that were identified during the review of vaccine records, 51% (n=169/307) were administered more than nine years before self-report. Although vaccine records were available from at least one source of care in all participants, approximately 25% (n=78/308) of participants had missing or unavailable vaccine records in one or more of their reported sources of care. Most missing records were due to the provider’s medical record retention policy (46%) or from not granting access (36%).

Self-reported immunization status using CASI had an accuracy of 84% (95% CI 81-89), a sensitivity of 89% (95% CI 82-94) and a specificity of 80% (95% CI 74-86). The positive and negative predictive values were 74% (95% CI 66-81) and 92% (95% CI 87-96), respectively. Among the 50 participants whose self-reported immunization status was discordant with the cumulative records of their medical providers, 74% (n=37/50) were due to overreporting immunization, and 26% (n=13/50) were due to underreporting, as shown in Table 3.

**Table 3:** Performance of self-reported immunization status for the HPV vaccine.

Self-report	Provider-verified		Total
	Ever immunized	Not immunized	
Ever immunized	107	37	144
Not immunized	13	151	164
Total	120	188	308

Accurate immunization status by self-report was not associated with the specific characteristics of the participants (Table 4). Of the 107 women who accurately reported having been immunized, 65% (n=70/107) also accurately reported the total number of doses they had received, and 35% (n=37/107) accurately reported the year in which they had received the first dose of the vaccine. Public insurance consisted of Medicare, Medicaid, HUSKY, Indian Health Service, or military insurance. The *P* values used unadjusted odds ratio for associations between characteristics of the subjects and accuracy of self-reporting of immunization with the HPV vaccine using logistic regression (missing/unknown observations were excluded).

**Table 4:** Association between characteristics of subjects and the accuracy of self-report.

Demographics		Accurate immunization status	
		OR^a^ (95% CI)	*P* value
Age, years		1.02 (0.93-1.13)	.63
**Race/ethnicity**			
	Non-Hispanic white	Reference	
	Non-Hispanic black	0.83 (0.36-1.89)	.66
	Hispanic	0.76 (0.35-1.76)	.51
	Non-Hispanic other/multi-race	0.47 (0.17-1.30)	.15
Publicly insured		1.69 (0.77-3.59)	.18
Some college education		1.38 (0.67-2.82)	.37
Annual income of <US $50,000		0.58 (0.30-1.19)	.13

^a^OR: odds ratio.

Many participants (86%; n=268) were able to respond correctly to half of the questions about their knowledge of the HPV vaccine. Knowledge of the HPV vaccine was similar among participants whose self-reported immunization status was accurate, and those whose self-reported immunization status was discordant with that in the medical records (Table 5). The *P* values were calculated using the chi-squared test.

**Table 5:** Association between baseline knowledge of HPV and accuracy of self-reporting.

Correctly identified	Self-report		
	Accurate, %	Inaccurate, %	*P* value
HPV^a^ is an STD^b^	82	78	.53
HPV is common	88	84	.44
HPV affects both men and women	89	88	.88
HPV infections peak in 20s and 30s	21	16	.46
HPV causes genital warts	67	66	.84
Average number correct	69	66	.36^c^

^a^HPV: human papillomavirus.

^b^STD: sexually transmitted disease.

^c^Calculated using a two-sample, two-tailed *t* test with equal variances.

### Sensitivity Analyses

The results of the sensitivity analyses are shown in Table 6. Differences in overall accuracy between the primary analyses and the sensitivity analyses were <5%. The accuracy of self-report was similar between cases and matched controls. Excluding the 42 participants who were uncertain about their prior immunization (those who responded “I don’t know” when asked if they had ever been immunized), there was also no substantial change to the overall accuracy of self-reported immunization status (85%; 95% CI 80-89).

**Table 6:** Sensitivity analyses: differences in overall accuracy.

Sensitivity models	Accuracy, %	95% CI, %	Difference, %
Included in performance analysis, n=308	84	79-88	Reference
Cases, n=107	86	78-92	–2.6
Controls, n=201	83	77-88	0.9
Only if complete medical records, n=232	87	82-91	–3.9

## Discussion

### Primary Findings

Ascertaining whether a person has ever been immunized with the HPV vaccine can be challenging and resource-intensive. In this study, we assessed the use of a computer-assisted self-interviewing instrument to ascertain HPV-vaccine immunization status by self-report with an instrument that was easy to access, user-friendly, and optimized for mobile devices. We found that this approach was feasible and reasonably accurate (84%) in a clinical research setting. Using this instrument, our research team was able to correctly identify 89% of women who had previously been immunized with the HPV vaccine in a relatively short period. In a setting of moderate coverage as in the United States (39% immunized) [2], we found that a negative test (not-immunized or unsure if immunized by self-report) was highly predictive of a patient who had never been immunized (negative predictive value=92%).

Several valuable lessons were learned through the testing and implementation of this computer-assisted self-interviewing instrument. First, we found that this approach was feasible and acceptable to adult women enrolled in a clinical research project. An overwhelming majority of participants favored completing the survey on their device rather than scheduling an in-person meeting or waiting for a mail-in questionnaire. Second, we learned that by allowing participants to complete the survey independently, the time our staff would have spent conducting interviews and entering survey responses could be diverted to other important tasks. Third, we found that acceptability of the survey was high, and the overall proportion of participants who stopped answering questions after starting the survey was low.

Although several studies have previously assessed the accuracy of self-reported immunization with the HPV vaccine in adults, all have done so using either telephone or in-person interviews [18-26]. The range in accuracy of self-report found in these previous studies has been wide (59-90%). Our study differed from these previous attempts to measure accuracy of self-report by using a novel computer-assisted self-interviewing instrument that may remove the perceived time pressures to respond, and that provides respondents with an enhanced sense of privacy. Moreover, our study is the only one that compared the results of self-reporting to the immunization status determined from an exhaustive review of vaccine records at multiple sources of care.

Finally, we found that in the process of verifying self-reporting, a substantial amount of time and resources were spent contacting health care providers who either were not always willing to participate or did not always possess complete vaccine records. Thus, it is possible that had we used our computer-assisted self-interviewing instrument alone, we could have estimated the participant’s HPV vaccine immunization status in a much less time- and resource-intensive manner without substantially sacrificing accuracy. Although our study did not test these potential gains in efficiency, our data suggest that these methods warrant further investigation. Identifying a data-collection strategy that is both accurate and efficient would be an essential public health contribution as even small improvements in the way we collect data about prior immunization could substantially reduce costs and facilitate the study of the HPV vaccine in this under-immunized population.

### Potential Limitations

This study has some potential limitations. First, it used data from a sample of adult women who were participating in a case-control study. Thus, bias may have been introduced in the selection of subjects. However, there was very little difference in the accuracy of self-reporting between cases and controls, which suggests that combining the groups is unlikely to have led to bias [27-29]. Second, the measure we used as a gold standard (all reported sources of care) may not have captured all doses of the HPV vaccine, as some women may not have correctly recalled all prior sources of care, and some providers had incomplete vaccine records. However, results were largely unchanged when we excluded women for whom all records could not be reviewed. Third, an inherent limitation to computer-assisted self-interviewing is the lack of any participant-researcher interaction, which may lead to incorrect responses if any questions are unclear. However, to reduce any risk of this potential limitation, we tested and refined our instrument before deployment to ensure the clarity of questions and ease of use of our instrument.

### Conclusions

Accurately determining prior immunization with the HPV vaccine can be challenging and resource-intensive. Electronic data collection systems that utilize computer-assisted self-interviewing methodologies have been increasingly used in clinical research and offer a promising approach for ascertaining HPV vaccine immunization history. Our experience implementing a computer-assisted self-interviewing instrument suggests that it is a reasonably accurate method to ascertain immunization status by self-reporting, it is acceptable to adult women in a research setting, and it is feasible to implement.

## Appendix

Multimedia Appendix 1 Survey questions.

## Abbreviations

HPV: human papillomavirus
HPV-VE: human papillomavirus vaccine effectiveness
NIH: National Institutes of Health
