# Supplementary material for: Feasibility and Accuracy of a Computer-Assisted Self-Interviewing Instrument to Ascertain Prior Immunization With Human Papillomavirus Vaccine by Self-Report: Cross-Sectional Analysis
Source: JMIR Med Inform. 2020 Jan 22;8(1):e16487. doi: 10.2196/16487 (PMC7003116; doi:10.2196/16487)
Supplement: Multimedia Appendix 1 [file medinform_v8i1e16487_app1.docx]

Appendix

Table A1. Survey Questions

| **Questions** | | **Response options** |  |
| --- | --- | --- | --- |
| **Immunization History** | | |  |
| Have you ever received the human papillomavirus vaccine? (also known as Gardasil or HPV shot or cervical-cancer vaccine) | | - Yes - No - I don't know |  |
| How many doses of the HPV vaccine have you received? (also known as Gardasil or HPV shot or cervical cancer vaccine) | | - 1 dose - 2 doses - 3 doses - More than 3 doses - I do not know |  |
| Please list the name and address of the medical practice where you received human papillomavirus vaccines (please provide as much information as you can) | | Free text |  |
| Please list the date (month and year) when you received human papillomavirus vaccines (please provide as much information that you can recall) | | Free text |  |
| **Prior sources of care** | | |  |
| Please provide the name of your pediatrician office and office location (if you went to multiple practices please include all of them). | | Free text |  |
| Please provide the name of your gynecologist's office and office location (if you went to multiple practices please include all of them). | | Free text |  |
| Please provide the name and office location of any medical provider that you visited since 2006 (other than the previously listed pediatrician and gynecologist) | | Free text |  |
| **Sociodemographic** | | |  |
| What is the highest grade or year of school you completed? | - Never attended school or only attended kindergarten - Grades 1 through 8 (Some elementary) - Grades 9 through 11 (Some high school) - Grade 12 or GED (High school graduate) - College 1 year to 3 years (Some college or technical school) - College 4 years or more (College graduate) - I don’t know | | |
| What was the average annual income of your household from all sources | - Less than $10,000 - $10,000 to less than $20,000 - $20,000 to less than $30,000 - $30,000 to less than $50,000 - $50,000 to less than $70,000 - $70,000 to less than $100,000 - More than $100,000 - I don’t know - I do not wish to answer | | |
| Are you Hispanic/Latino? | - Yes - No - I don't know - I do not wish to answer | | |
| What is your race? (Select one or more responses.) | - White - Black or African American - Native Hawaiian or Other Pacific Islander - American Indian or Alaska Native - Asian - Other - I don't know - I do not wish to answer | | |

**Table A2. Baseline Human Papillomavirus Knowledge Questions**

| HPV infection is not sexually transmitted. | - True - False |
| --- | --- |
| HPV infection is relatively uncommon. | - True - False |
| Who can become infected with HPV? | - Men - Women - Both women and men |
| Most HPV infections occur in women during their 20's and 30's | - True - False |
| HPV causes genital warts | - True - False |
